# High-Throughput Chemical Screening Identifies Compounds that Inhibit Different Stages of the *Phytophthora agathidicida* and *Phytophthora cinnamomi* Life Cycles

**DOI:** 10.3389/fmicb.2017.01340

**Published:** 2017-07-19

**Authors:** Scott A. Lawrence, Charlotte B. Armstrong, Wayne M. Patrick, Monica L. Gerth

**Affiliations:** Department of Biochemistry, University of Otago Dunedin, New Zealand

**Keywords:** oomycetes, *Agathis australis*, kauri dieback, avocado root rot, phenotype microarray, zoospore, high-throughput screening

## Abstract

Oomycetes in the genus *Phytophthora* are among the most damaging plant pathogens worldwide. Two important species are *Phytophthora cinnamomi*, which causes root rot in thousands of native and agricultural plants, and *Phytophthora agathidicida*, which causes kauri dieback disease in New Zealand. As is the case for other *Phytophthora* species, management options for these two pathogens are limited. Here, we have screened over 100 compounds for their anti-oomycete activity, as a potential first step toward identifying new control strategies. Our screening identified eight compounds that showed activity against both *Phytophthora* species. These included five antibiotics, two copper compounds and a quaternary ammonium cation. These compounds were tested for their inhibitory action against three stages of the *Phytophthora* life cycle: mycelial growth, zoospore germination, and zoospore motility. The inhibitory effects of the compounds were broadly similar between the two *Phytophthora* species, but their effectiveness varied widely among life cycle stages. Mycelial growth was most successfully inhibited by the antibiotics chlortetracycline and paromomycin, and the quaternary ammonium salt benzethonium chloride. Copper chloride and copper sulfate were most effective at inhibiting zoospore germination and motility, whereas the five antibiotics showed relatively poor zoospore inhibition. Benzethonium chloride was identified as a promising antimicrobial, as it is effective across all three life cycle stages. While further testing is required to determine their efficacy and potential phytotoxicity *in planta*, we have provided new data on those agents that are, and those that are not, effective against *P. agathidicida* and *P. cinnamomi*. Additionally, we present here the first published protocol for producing zoospores from *P. agathidicida*, which will aid in the further study of this emerging pathogen.

## Introduction

Species in the genus *Phytophthora* (from the Greek for “plant-destroyers”) are among the most serious threats to native plants and horticultural species alike, with a global economic impact estimated in the billions of dollars per annum (Erwin and Ribeiro, 1996). While they superficially resemble filamentous fungi, *Phytophthora* are actually oomycetes and therefore more closely related to diatoms and brown algae in the stramenopiles (Gunderson et al., 1987; Thines, 2014). *Phytophthora* live in plant tissues, soil, or water, and under favorable conditions of temperature, moisture, and nutrition produce sporangia, followed by the release of motile zoospores, allowing infection to spread rapidly among host plants (Judelson and Blanco, 2005).

Two species of particular importance in New Zealand are *Phytophthora agathidicida* and *Phytophthora cinnamomi. P. agathidicida* (formerly *Phytophthora* taxon agathis or PTA) is a root and collar/stem canker pathogen responsible for dieback in kauri (*Agathis australis*), an iconic tree that is native to New Zealand (Beever et al., 2009; Weir et al., 2015). Kauri is a keystone species that has a profound influence on the surrounding soil, canopy, and biodiversity (Ecroyd, 1982; Wyse et al., 2014). Kauri dieback was first recognized over 40 years ago (Gadgil, 1974) and is continuing to spread through the forests of northern New Zealand, rapidly killing trees of all ages and sizes (Beever et al., 2009). Currently, there are no established treatment or control options for *P. agathidicida*, although the use of phosphite as a potential treatment for infected trees is being explored (Horner and Hough, 2013; Horner et al., 2015).

*Phytophthora cinnamomi* is a generalist pathogen capable of infecting thousands of different host plants in wildlands, horticulture and commercial nurseries, resulting in root rot and often death of the host plant (Erwin and Ribeiro, 1996). In New Zealand, the most economically important host is avocado (*Persea americana*). Globally, its principal food crop hosts are avocado and pineapple, but it is also an important pathogen of ecologically significant native plants throughout the temperate regions of the world. Some of the greatest plant losses are in areas with a Mediterranean climate including parts of the United States, Australia, Mexico, and the Iberian Peninsula (Burgess et al., 2017). It also infects a range of ornamental trees and shrubs and is readily spread through the movement and out-planting of nursery stock (Erwin and Ribeiro, 1996). Disease caused by *P. cinnamomi* has significant economic impacts on forestry and horticulture; in California alone, the annual loss from *P. cinnamomi* infections in avocado groves is US$30 million (Erwin and Ribeiro, 1996). *P. cinnamomi* has been called the “biological bulldozer” for its capacity to destroy plants (Carter, 2004), and it was recently identified as one of the top 10 oomycete pathogens, based on its scientific and economic importance (Kamoun et al., 2015).

Exacerbating the effects of *Phytophthora* infection is the fact that these species are notoriously difficult to control. While *Phytophthora* share many traits with the true fungi, they are phylogenetically distinct enough to lack many of the canonical molecular targets of labeled fungicides (Rolando et al., 2016). For example, *Phytophthora* species lack the biosynthetic pathways for ergosterol and a chitin-based cell wall. The development of resistance is also an ongoing problem (Cohen and Coffey, 1986; Parra and Ristaino, 2001; Gisi and Sierotzki, 2008; Miao et al., 2016). Once an area becomes infected, eradication can require chemical treatment, physical barriers to prevent disease spread, and destruction of host plants (Dunstan et al., 2010). Environmental toxicity of chemical treatments and the logistics of application further complicate disease management and eradication efforts.

In this study, we have sought to identify anti-oomycete compounds that inhibit mycelial growth, zoospore germination, and/or zoospore motility in *P. agathidicida* and *P. cinnamomi*, as a first step toward development of more effective control agents. By using phenotype microarray (PM) plates (Bochner et al., 2001) as sources of potential antimicrobials, we screened 120 known antimicrobial chemicals in a high-throughput fashion. Here, we explore the chemical susceptibility of the emerging pathogen, *P. agathidicida*, and compare its susceptibility profile to these chemicals with the more well-characterized species, *P. cinnamomi.*

## Materials and Methods

### Routine Culturing

*Phytophthora agathidicida* isolate NZFS 3770 and *P. cinnamomi* isolate NZFS 3910 were sourced from Scion (Rotorua, New Zealand). The two species were routinely cultured at 22°C in darkness on clarified 20% V8-juice agar (V8A). For antibiotic susceptibility testing using PM plates (Biolog Inc, CA, United States), isolates were cultured on potato-dextrose agar (PDA; *P. agathidicida*) or cornmeal agar (CA; *P. cinnamomi*) (Becton, Dickinson & Co, NJ, United States). EC_50_ assays were carried out using CA for both species.

### Anti-oomycete Compound Screening

The activities of potential anti-oomycete compounds were tested using a modified version of the Kirby–Bauer disk diffusion assay (Bauer et al., 1966). Test compounds were prepared by adding 20 μL of sterile water to each well of a PM plate. Each plate (PM 21D, 22D, 23A, 24C, and 25D) contains 24 different compounds at four different concentrations. Five microliters of each resuspended compound were added to sterile 6 mm filter disks (Whatman number 1 paper). After sufficient drying, the four filter disks containing a single test compound at different concentrations were placed at regular intervals around the edges of a PDA or CA plate, along with a control disk (sterile water). Each plate was then inoculated by placing a 3 mm agar plug of mycelium (taken from the leading edge of an actively growing mycelial mat) in the center. Each plate was incubated at 20°C in the dark and radial growth was measured after 5 days (*P. cinnamomi*) or 7 days (*P. agathidicida*). Three biological replicates (from independently grown cultures) were performed for each compound screened. Any compound that reproducibly gave zones of inhibition that were greater than the control disk and were dose-dependent was scored as a positive.

### Determination of EC_50_ Values for Inhibition of Mycelial Growth

The EC_50_ values for radial growth inhibition were determined for each compound that inhibited *Phytophthora* growth in the disk diffusion assays. CA was amended with eight twofold dilutions of the compound of interest, with concentrations ranging from 0.125 to 512 μg/ml. Concentration ranges were chosen based on preliminary growth inhibition assays and differed among compounds. Control plates (CA only) were included for each compound tested, and three biological replicates were performed for each compound and concentration. Each plate was inoculated by placing a 3 mm agar plug of mycelium (taken from the edge of an actively growing mycelial mat) in its center; plates were then incubated at 20°C for 4 days (*P. cinnamomi*) or 6 days (*P. agathidicida*). Two perpendicular measurements were taken for each plate and averaged, and the size of the agar plug was subtracted to obtain the final measurement of radial growth. Measurements from test compound plates were subtracted from control plate measurements and converted to inhibition percentages. To estimate EC_50_ values, compound concentrations were log-transformed, and non-linear regression with curve fitting (by least squares) was carried out using GraphPad Prism version 6.0. All chemicals were purchased from Sigma-Aldrich (MO, United States) except for copper sulfate pentahydrate (Ajax Chemicals, Sydney, Australia) and kanamycin sulfate (Applichem GmbH, Darmstadt, Germany).

### Zoospore Germination and Motility

In order to produce sporangia in *P. cinnamomi* and *P. agathidicida*, approximately 2 cm^2^ of agar from the edge of an actively growing mycelial mat was excised and transferred to a Petri dish containing a 1:10 dilution of carrot broth (*P. agathidicida*) or clarified 20% V8 broth (*P. cinnamomi*), cut into ∼1 mm^2^ pieces and then incubated at 25°C in darkness for 24 h. The following day, the broth was removed and each dish was washed four times in sterile Chen–Zentmyer salt solution (for *P. cinnamomi*; Chen and Zentmyer, 1970) or sterile pond water (for *P. agathidicida*) for 45 min at a time on the bench (*P. agathidicida*) or on a rocking table (*P. cinnamomi*), followed by overnight incubation (without rocking) at 22°C under light. Following sporangium production, zoospore release was induced by removing the liquid and washing each dish three times with sterile Milli-Q water that had been cooled to 4°C. Each wash was for 20 min, with the first two at room temperature and the final wash at 4°C. Wash volumes were 15 ml per dish for the first two washes, and 10 ml for the final wash. Following the final wash, dishes were returned to room temperature for 30–90 min until sufficient numbers (a final concentration of approximately 1 × 10^3^ zoospores/ml) of zoospores had been released.

To assess the effect of each putative anti-oomycete compound on zoospore germination, 200 μl of zoospore suspension were spread on triplicate water agar plates amended with the test compounds. Concentrations of the compounds were as for the mycelial growth inhibition assays, and ranged from 0.125 to 512 μg/ml. Plates were incubated for 12–16 h, and 100 zoospores per plate were counted under a Nikon C-DS dissecting microscope at 40× magnification to determine the percentage that had germinated. Spores were defined as having germinated if the germ tube length was at least twice as long as the spore diameter, as described previously (Liu et al., 2014). The level of germination inhibition was calculated by subtracting the percent germinated at each concentration from that of three control (water agar only) plates.

Zoospore motility assays were based on a method described previously (Hu et al., 2007). One milliliter of zoospore suspension (approximately 1,000 zoospores) was added to triplicate wells in a 24-well plate containing 10 μl of the test compound solution at different concentrations, resulting in five twofold dilutions of the test compound. Control wells were amended with an equal volume of sterile water. Zoospores were kept at room temperature, and observed with the dissecting microscope to determine the time required for all zoospore motility to stop. Observations were made at 5 min intervals for the first hour, then at 30 min intervals until motility had ceased. Wells that had no motile zoospores by the time of the first observation (5 min post-treatment) were scored as 0 (i.e., complete inhibition). Subsequent loss of motility was calculated by dividing the length of time zoospores remained motile by the average motility time in the controls.

## Results

### High-Throughput Screening for Anti-oomycete Compounds

**FIGURE 1:**
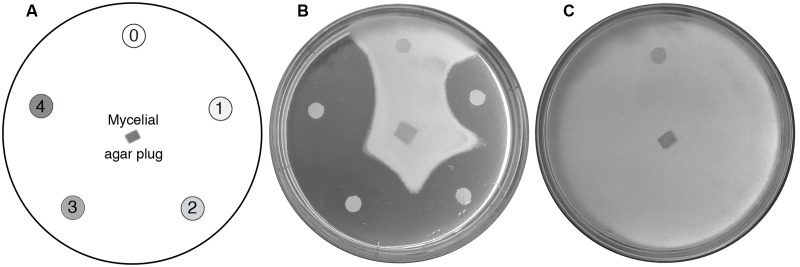
High-throughput screening for anti-oomycete compounds. **(A)** Diagram showing the standard test arrangement. An agar plug of mycelium is used to inoculate an agar plate with disks saturated either with water (control; marked 0) or increasing concentrations of a test compound (in a clockwise direction, marked 1–4). **(B)** Representative results from a typical experiment. Here, *P. agathidicida* mycelial growth is inhibited in the presence of increasing concentrations of paromomycin. **(C)** Control plate showing normal growth of *P. agathidicida* in the absence of test compounds.

The activities of potential anti-oomycete compounds were tested using a disk diffusion assay (Bauer et al., 1966), where the zone of inhibition of mycelial growth around filter disks containing the test compounds was assessed (**Figure 1**). Each species was screened against a panel of Biolog PM plates. In total, the panel of five PM plates contained 120 potential antimicrobial compounds, with each compound present at four concentrations. The panel contained common anti-yeast and antifungal antibiotics, as well as other types of chemicals with antimicrobial activity such as metals, detergents, and inhibitors. The complete list of compounds tested is available in Supplementary Table 1.

Of the 16 anionic toxins tested, only one (arsenite) had an inhibitory effect. Similarly, only two of the 15 cationic toxins tested, copper (II) chloride and copper (II) sulfate, showed inhibitory effects. None of the chelating agents tested (including dipyridyl, EDTA, and EGTA) showed any inhibitory effects.

Several common antibiotics successfully inhibited growth, particularly at higher concentrations. The majority were aminoglycosides (hygromycin B, kanamycin, neomycin, paromomycin, and tobramycin), which act by inhibiting protein synthesis. Chlortetracycline (a tetracycline antibiotic which also inhibits protein synthesis) and D-cycloserine (an amino acid derivative which inhibits pyridoxal 5′-phosphate dependent enzymes) also inhibited mycelial growth. Of the antibiotics identified here, only neomycin and paromomycin have been previously reported to have anti-oomycete activity against other *Phytophthora* species (Lee et al., 2005).

In addition to antibiotics, the PM screen included seven common antifungals: three triazoles (fluconazole, myclobutanil, propiconazole), an imidazole (miconazole), a polyene antimycotic (nystatin), a nucleoside analog (5-fluorocytosine) and cycloheximide. None of these showed any inhibitory effects.

Benzethonium chloride, a quaternary ammonium salt often used as a disinfectant, showed strong inhibition of *P. agathidicida* mycelial growth, and a low level of inhibition against *P. cinnamomi*.

Thallium (I) acetate and sodium arsenite both showed promising inhibitory effects; however, their non-selective toxicity (Hughes, 2002; Lennartson, 2015) makes them unsuitable for use in the environment so they were not tested further. Similarly, fluorodeoxyuridine (an oncology drug) and trifluoperazine (an antipsychotic) inhibited mycelial growth, but were excluded from further analysis due to their unsuitability for use in the real-world treatment of oomycete infections.

### Mycelial Growth Inhibition

**FIGURE 2:**
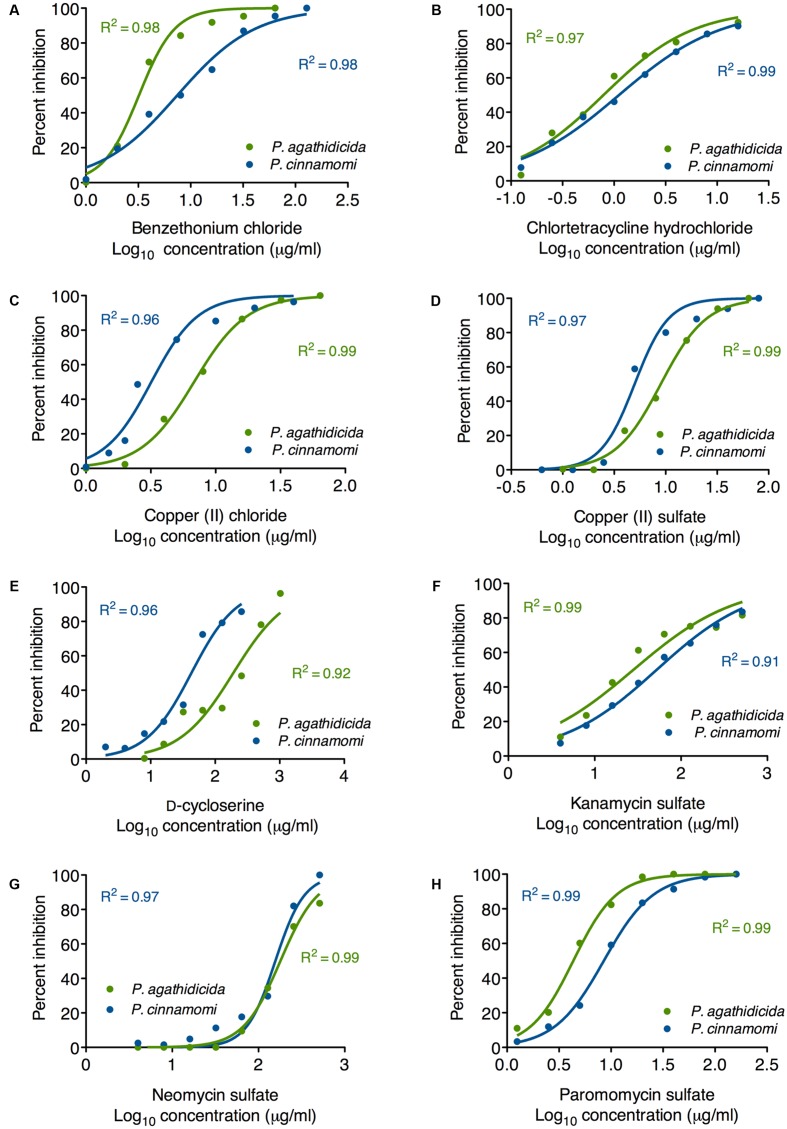
Mycelial growth inhibition curves for *P. agathidicida* (green) and *P. cinnamomi* (blue). Each panel **(A–H)** shows the inhibition curves for a single compound, as identified in the label on the *x*-axis. Data points show the mean of triplicate inhibition assays.

Based on the results of our qualitative high-throughput screen, we selected the eight most promising inhibitors for more quantitative testing. To begin, we determined EC_50_ values for inhibition of mycelial growth. This is important, because mycelial growth assays approximate both systemic growth within the plant, and across root-grafts between plants (i.e., root-to-root contact). Regression analysis resulted in well-supported inhibition curves, with all *R*^2^ values >0.9 (**Figure 2**). The calculated EC_50_ values for all compounds are presented in **Table 1**. These values varied widely among the compounds tested, from 0.79 to 180 μg/ml.

**Table 1:** EC_50_ values for inhibition of mycelial growth.

	*P. agathidicida* EC_50_ (μg/ml)	*P. cinnamomi* EC_50_ (μg/ml)
Benzethonium chloride	3.2 (2.3-3.5)	7.3 (6.5-8.3)
Chlortetracycline hydrochloride	0.79 (0.69-0.90)	1.1 (1.0-1.2)
Copper (II) chloride	6.8 (6.5-7.2)	3.2 (2.9-3.6)
Copper (II) sulfate	9.0 (8.4-9.6)	5.0 (4.5-5.5)
D-cycloserine	190 (150-240)	43 (37-49)
Kanamycin sulfate	28 (22-35)	52 (48-57)
Neomycin trisulfate	180 (170-190)	160 (140-170)
Paromomycin sulfate	4.3 (4.0-4.6)	8.5 (8.2-8.9)


*Numbers in parentheses are 95% confidence intervals, determined from three independent experiments.*

The most effective inhibitor of mycelial growth in both *Phytophthora* species was chlortetracycline hydrochloride, with EC_50_ values of 0.79 and 1.1 μg/ml in *P. agathidicida* and *P. cinnamomi*, respectively. Benzethonium chloride was also an effective inhibitor, though more so for *P. agathidicida*, as was seen in the initial high-throughput screen. Both of the copper compounds tested had relatively low EC_50_ values, and were more effective against *P. cinnamomi*. EC_50_ values for the aminoglycoside antibiotics varied widely, with paromomycin by far the most effective. D-cycloserine was a relatively ineffective inhibitor, although it showed the greatest species specificity of any compound tested (almost fivefold more effective against *P. cinnamomi* than *P. agathidicida*).

### Zoospore Germination Inhibition

In addition to mycelial growth, a useful antimicrobial agent could also inhibit one or more of the other stages in the *Phytophthora* life cycle (see, for example, Mircetich, 1970 and Judelson and Blanco, 2005). The first step in initiating a new infection is the encystment and germination of a zoospore on the host plant (Judelson and Blanco, 2005). We tested the ability of our eight candidate antimicrobials to inhibit zoospore germination, following a previous protocol (Liu et al., 2014).

As no previous studies have reported laboratory methods for producing *P. agathidicida* zoospores, several variations on the standard *P. cinnamomi* protocol were trialed in order to maximize the number of sporangia formed and zoospores released. It was found that excised pieces of mycelial mats grew best when grown in diluted, clarified carrot broth (Erwin and Ribeiro, 1996) rather than V8 broth. Sporangia production was increased when the subsequent washing steps were carried out using sterile (filtered and autoclaved) pond water (pH ∼8.5) rather than the salt solution used for *P. cinnamomi*. The chemical composition of the pond water used can be found in Supplementary Table 2. Pond water samples from two sources (Rotorua, North Island, New Zealand and Dunedin, South Island, New Zealand) were trialed, with no noticeable differences in sporangium numbers. Adjusting the pH of the pond water up or down, to values between 5 and 10, reduced sporangia production, emphasizing that unadjusted pond water was optimal. As the mycelial mats of *P. agathidicida* were less adherent than those of *P. cinnamomi*, carrying out the wash steps on a rocking table tended to dislodge the mats and resulted in decreased numbers of sporangia; therefore wash steps were carried out without rocking. Methods for inducing zoospore release were as for *P. cinnamomi*, though *P. agathidicida* often required a longer final incubation step at room temperature before zoospores began to be released.

**FIGURE 3:**
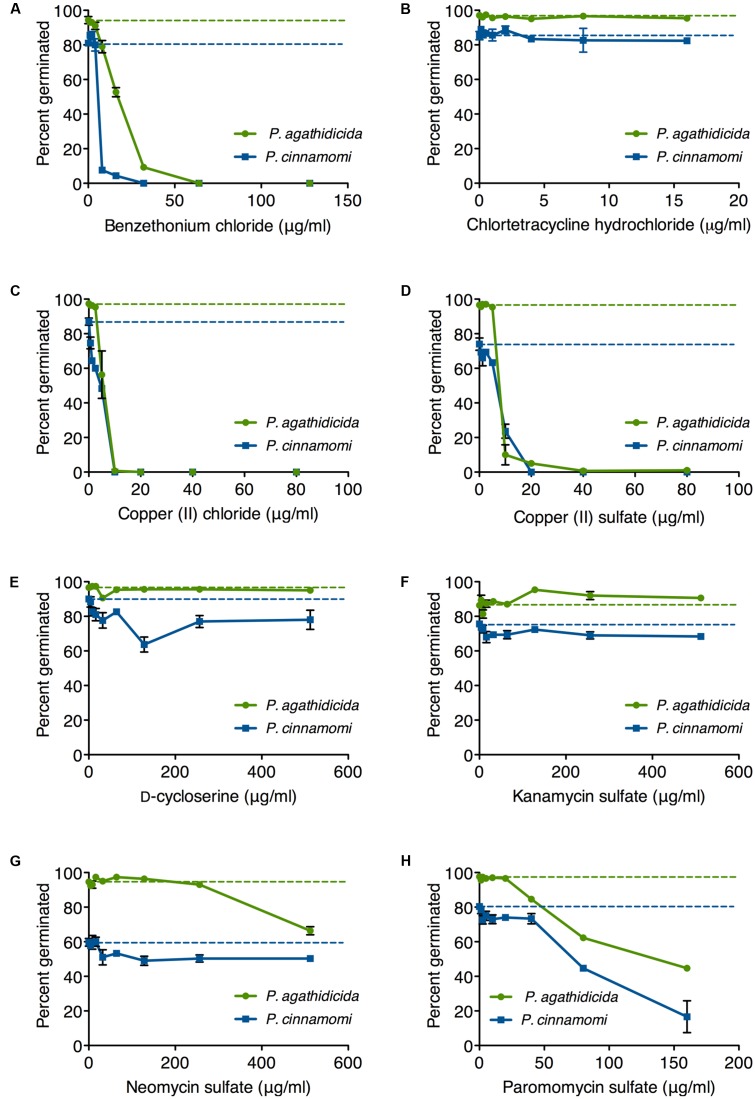
Inhibition of zoospore germination in *P. agathidicida* (green) and *P. cinnamomi* (blue). Each panel **(A–H)** shows the inhibition curves for a single compound, as identified in the label on the *x*-axis. Dashed lines represent the germination rates on unamended agar. Data points are mean values from triplicate germination assays. Error bars are ± standard error of the mean. Where error bars are not visible, they are smaller than the symbol.

Germination rates on unamended agar plates ranged from 85 to 98% for *P. agathidicida* and 57–91% for *P. cinnamomi* (dashed lines in **Figure 3**). Germination of *P. agathidicida* zoospores was inhibited to a greater or lesser extent by five of the eight compounds tested (**Figure 3**), with the strongest inhibitors being benzethonium chloride (**Figure 3A**), copper (II) chloride (**Figure 3C**), and copper (II) sulfate (**Figure 3D**). All of these compounds inhibited germination completely at concentrations ranging from 10 to 80 μg/ml, with the two copper salts proving the most effective. On the other hand, neomycin and paromomycin only effected partial inhibition of germination at the highest concentrations tested (**Figures 3G,H**). The other antibiotics (chlortetracycline, D-cycloserine and kanamycin) did not inhibit germination of *P. agathidicida* zoospores.

The pattern was broadly similar for inhibiting germination of *P. cinnamomi* zoospores (**Figure 3**). As with *P. agathidicida*, the most effective inhibitors were benzethonium chloride (**Figure 3A**), copper chloride (**Figure 3C**), and copper sulfate (**Figure 3D**), all of which prevented germination completely at concentrations between 10 and 80 μg/ml. Paromomycin was the most effective of the antibiotics, although it only reduced the germination rate by fourfold (**Figure 3H**).

### Zoospore Motility Inhibition

A third way of disrupting *Phytophthora* pathogenicity is to inhibit zoospore motility. Once released from sporangia, zoospores exhibit chemotaxis toward the roots of a new host plant, and, in the case of *P. cinnamomi*, can swim at approximately 0.5 m/h through water (Allen and Newhook, 1973; Allen and Harvey, 1974). Rendering them immotile would offer a novel containment approach that prevents spread from infected trees.

In control experiments, the untreated zoospores of *P. agathidicida* and *P. cinnamomi* remained motile in sterile water for approximately 17 and 20 h on average, respectively. We tested our set of eight anti-oomycete compounds over a range of concentrations. In general, we observed dose dependent responses, with higher compound concentrations leading to faster zoospore encystment. The only exception was D-cycloserine, which had no effect on the motility of *P. cinnamomi* zoospores at any concentration tested.

In order to quantify the effectiveness of the compounds, we compared the lowest concentration of each that was required to cause complete loss of motility within 5 min of treatment. The concentration required for this rapid inhibition varied widely among compounds (**Table 2**). Copper chloride, copper sulfate, and benzethonium chloride proved the most effective, with concentrations ≤0.1 μg/ml causing almost immediate loss of motility. At the other extreme, 256 μg/ml D-cycloserine was required to effect such immediate inhibition of *P. agathidicida* zoospore motility. Similarly, at the highest concentration tested (8 μg/ml), kanamycin failed to inhibit motility within 5 min. Instead, it took 25 min for *P. agathidicida* zoospores to lose motility at this concentration of kanamycin, and 60 min for *P. cinnamomi* zoospores. The general trends observed for both *Phytophthora* species in response to the eight compounds were similar, although *P. agathidicida* zoospores were over an order of magnitude more sensitive to neomycin than *P. cinnamomi* (**Table 2**).

**Table 2:** Minimum concentrations of anti-oomycete compounds (in μg/ml) required to cause zoospores to become immotile within 5 min of treatment.

	*P. agathidicida*	*P. cinnamomi*
Benzethonium chloride	0.1	0.1
Chlortetracycline hydrochloride	16	8
Copper (II) chloride	0.025	0.05
Copper (II) sulfate	0.025	0.05
D-cycloserine	256	–
Kanamycin sulfate	–	–
Neomycin trisulfate	0.25^∗^	4
Paromomycin sulfate	0.8	1.6


*^∗^This was the lowest concentration tested, therefore the minimum concentration required for immediate motility loss may be lower.*

## Discussion

This study provides the first comprehensive screening data on dozens of potential anti-oomycete compounds, for their effectiveness against two species of *Phytophthora*. In the case of *P. cinnamomi*, we have extended comparable early work on screening 13 fungicides (Smith, 1979) to incorporate a significantly broader range of compounds. For the emerging pathogen *P. agathidicida*, we have reported the first such high-throughput screen. By assessing effectiveness against three different stages of the *Phytophthora* life cycle, we have also explored new opportunities for control of these destructive organisms.

Of the 120 compounds screened, eight showed anti-oomycete activity against both *Phytophthora* species. *P. agathidicida* is an emerging pathogen, and none of the inhibitory compounds found here have been reported previously. The EC_50_ values for mycelial growth inhibition were all within an order of magnitude between the two species, suggesting that similar control options may be available for *P. agathidicida* as those that are used for *P. cinnamomi*. However, further study of the various known isolates of *P. agathidicida* and *P. cinnamomi* will be necessary in order assess any potential differences in sensitivities.

Five of the eight anti-oomycete compounds identified in our screens were antibiotics that are more commonly associated with antibacterial activity. Antibiotics have been used since the 1950s to control bacterial diseases of various plants. However, with the growing threat of antibiotic resistance, this practice is the subject of debate (McManus et al., 2002; Tripathi and Cytryn, 2017; Wiles, 2017). The inhibitory abilities of the antibiotics identified in this study differed markedly between *Phytophthora* life cycle stages. This was particularly true of chlortetracycline, which was the most effective mycelial growth inhibitor in both species, but had no effect on zoospore germination rates and showed relatively poor inhibition of zoospore motility. Paromomycin was the only antibiotic tested here that inhibited all three life cycle stages, albeit with less efficacy in zoospores than the copper-containing compounds or benzethonium chloride. Overall, given the limited efficacy of these antibiotics against the different life cycle stages of *Phytophthora* and the potential for resistance to spread within and between species, our data suggest there is little potential for exploring these antibiotics as treatments for *Phytophthora* diseases, except perhaps as a last resort.

The most effective compounds across all three life cycle stages that we identified were copper salts and the quaternary ammonium salt benzethonium chloride. The identification of copper in our screens is unsurprising, as copper-based fungicides have traditionally been a mainstay of *Phytophthora* control (Keast et al., 1985; Howard et al., 1998; Gisi and Sierotzki, 2008). However, it is interesting to note that both copper chloride and copper sulfate were ∼2-fold less effective at inhibiting mycelial growth in *P. agathidicida* as compared to *P. cinnamomi*. In New Zealand, copper sprays have been used widely in agricultural, horticultural, and forestry settings to control a range of bacterial and fungal pathogens. For example, copper spraying has become a significant component of the kiwifruit industry’s spray program for providing protection against infection by *Pseudomonas syringae* pv. *actinidiae* (Cameron and Sarojini, 2014; Gould et al., 2015). Similarly, copper fungicides are widely used by the New Zealand forestry industry for control of dothistroma needle blight (Bulman et al., 2013) and they have recently been shown to be effective against *Phytophthora pluvialis* (Rolando et al., 2016). However, concerns around soil accumulation and toxicity limit the use of copper sprays in natural environments (Wightwick et al., 2008; Komarek et al., 2010; Kiaune and Singhasemanon, 2011).

The quaternary ammonium salt benzethonium chloride was also effective across all three life cycle stages, for both species we examined. It showed EC_50_ values for mycelial growth inhibition of <10 μg/ml, immediate loss of zoospore motility at 0.1 μg/ml, and complete inhibition of germination at 32 μg/ml (*P. cinnamomi*) or 64 μg/ml (*P. agathidicida*). To our knowledge, this is the first report of the effectiveness of benzethonium chloride against any *Phytophthora* species, although other quaternary ammonium compounds have been shown to be effective against *P. cinnamomi* (Noske and Shearer, 1985). For example, benzalkonium chloride is sold in Australia under the brand name Phytoclean and is designed for use in shoe wash stations and for equipment/tool washdown. While preliminary, our data suggest that benzethonium chloride may be similarly useful, for example, in the shoe wash stations and sanitation kits that are currently employed to prevent the spread of kauri dieback. Before implementation, future work will need to include studies on phytotoxicity and the potential to be used as a soil drench, as was done during the commercialization of Phytoclean.

While our work expands the range of compounds known to possess anti-oomycete activity toward *P. cinnamomi*, it is particularly interesting with respect to *P. agathidicida*. As this species is an emerging pathogen that was only formally described in 2015 (Weir et al., 2015), comparatively little research has been carried out on treatment options. To date, phosphorous acid (phosphite) is the only chemical that has been used on kauri trees affected by *P. agathidicida*, and while this has proved an effective treatment, some signs of phytotoxicity were present at higher treatment concentrations (Horner and Hough, 2013; Horner et al., 2015). The EC_50_ for mycelial growth inhibition by phosphite was 4 μg/ml (Horner and Hough, 2013), similar to the EC_50_ we measured for benzethonium chloride (3.2 μg/ml). It must be noted, however, that phosphite acts both by inhibiting pathogen growth and by inducing host defense responses, therefore *in vitro* assays may overestimate the dose required *in planta* (Erwin and Ribeiro, 1996). Furthermore, copper chloride, copper sulfate, and benzethonium chloride all inhibited *P. agathidicida* zoospore motility and germination at low concentrations. Future work will also explore their effects on other stages of the *P. agathidicida* life cycle, such as the formation of the chlamydospores, oospores, and hyphal aggregates that are produced inside the roots of the hosts, as these may be critical targets for localized disease eradication.

Clearly work remains to be done to combat the threat to kauri posed by *P. agathidicida*. In this study, we have provided a list of promising anti-oomycete compounds, as well as a protocol for laboratory production of *P. agathidicida* zoospores. Our results suggest avenues for further investigation, bearing in mind that numerous factors must be considered before employing chemical control to manage plant disease, including: the rate, method, and frequency of application; possible phytotoxicity; and the potential for development of resistance. Ultimately, we hope that this study will facilitate further research into this pathogen, thereby helping to prevent the devastation of some of New Zealand’s most iconic forests.

## Author Contributions

MG conceived this study. All authors participated in the experimental design, protocol development, and culturing of *Phytophthora*. SL and CA conducted all of the inhibition assays. All authors contributed to the writing and all authors have approved the final version.

## Conflict of Interest Statement

The authors declare that the research was conducted in the absence of any commercial or financial relationships that could be construed as a potential conflict of interest.
