# Supplementary material for: High-Throughput Chemical Screening Identifies Compounds that Inhibit Different Stages of the Phytophthora agathidicida and Phytophthora cinnamomi Life Cycles
Source: Front Microbiol. 2017 Jul 19;8:1340. doi: 10.3389/fmicb.2017.01340 (PMC5515820; doi:10.3389/fmicb.2017.01340)
Supplement: Supplementary file 1 [file Table_1.pdf]

Supplementary Material

High-throughput chemical screening identifies compounds that inhibit different stages of the *Phytophthora agathidicida* and *Phytophthora cinnamomi* life cycles

Scott A. Lawrence, Charlotte B. Armstrong, Wayne M. Patrick and Monica L. Gerth*

***Correspondence:** Corresponding Author: monica.gerth@otago.ac.nz

Supplementary Table 1. Compounds in the Biolog Phenotype Microarray plates used in this study.

**Type**	**Name**	**Biolog PM**	**Wells**
Anion	Potassium chromate	Plate PM24C	D5-D8
Anion	Sodium (*meta*)periodate	Plate PM23A	E5-E8
Anion	Sodium arsenite	Plate PM23A	E9-E12
Anion	Sodium benzoate	Plate PM24C	B9-B12
Anion	Sodium cyanate	Plate PM23A	F9-F12
Anion	Sodium cyanide	Plate PM23A	H5-H8
Anion	Sodium dichromate	Plate PM21D	D9-D12
Anion	Sodium fluoride	Plate PM24C	F9-F12
Anion	Sodium metaborate tetrahydrate	Plate PM23A	E1-E4
Anion	Sodium metasilicate	Plate PM24C	C5-C8
Anion	Sodium metavanadate	Plate PM22D	A5-A8
Anion	Sodium nitrite	Plate PM23A	G1-G4
Anion	Sodium orthovanadate	Plate PM23A	G5-G8
Anion	Sodium selenate	Plate PM23A	H1-H4
Anion	Sodium selenite	Plate PM21D	G1-G4
Anion	Sodium thiosulfate	Plate PM23A	H9-H12
Antibiotic	4-nitroquinoline-*N*-oxide	Plate PM25D	C9-C12
Antibiotic	Alexidine	Plate PM25D	D1-D4
Antibiotic	Aminacrine	Plate PM24C	A5-A8
Antibiotic	Apramycin sulfate	Plate PM24C	A1-A4
Antibiotic	Blasticidin hydrochloride	Plate PM24C	B1-B4
Antibiotic	Bleomycin	Plate PM22D	F9-F12
Antibiotic	Bleomycin	Plate PM22D	H1-H4
Antibiotic	Chloroalanine hydrochloride	Plate PM25D	B1-B4
Antibiotic	Clomiphene citrate	Plate PM25D	E9-E12
Antibiotic	D-cycloserine	Plate PM21D	F9-F12
Antibiotic	D,L-serine hydroxamate	Plate PM22D	C9-C12
Antibiotic	Dequalinium chloride	Plate PM23A	B5-B8
Antibiotic	Fluconazole	Plate PM24C	G9-G12

Antibiotic	Hygromycin B	Plate PM25D	D5-D8
Antibiotic	Ibuprofen	Plate PM25D	G9-G12
Antibiotic	Kanamycin monosulfate	Plate PM25D	B9-B12
Antibiotic	Miltefosine	Plate PM22D	C5-C8
Antibiotic	Neomycin	Plate PM21D	F5-F8
Antibiotic	Paromomycin	Plate PM22D	H5-H8
Antibiotic	Pentamidine isethionate	Plate PM24C	C9-C12
Antibiotic	Polymyxin B	Plate PM22D	D1-D4
Antibiotic	Tamoxifen	Plate PM24C	H5-H8
Antibiotic	Thialysine	Plate PM24C	D9-D12
Antibiotic	Thioridazine hydrochloride	Plate PM24C	B5-B8
Antibiotic	Tobramycin	Plate PM25D	A5-A8
Antibiotic	Triclosan	Plate PM22D	B9-B12
Antibiotic	Trifluoperazine	Plate PM21D	G9-G12
Antibiotic	Zaragozic acid A	Plate PM24C	A9-A12
Cation	Aluminum sulfate	Plate PM24C	G5-G8
Cation	Ammonium sulfate	Plate PM23A	A9-A12
Cation	Cadmium chloride hydrate	Plate PM23A	B1-B4
Cation	Chromium (III) chloride hexahydrate	Plate PM23A	D1-D4
Cation	Cobalt (II) chloride hexahydrate	Plate PM23A	D5-D8
Cation	Copper (II) sulfate	Plate PM21D	F1-F4
Cation	Cupric chloride dihydrate	Plate PM23A	D9-D12
Cation	Dodecyltrimethyl ammonium bromide	Plate PM21D	B5-B8
Cation	Lithium chloride	Plate PM22D	F5-F8
Cation	Magnesium chloride	Plate PM21D	E9-E12
Cation	Manganese (II) chloride	Plate PM21D	E5-E8
Cation	Nickel chloride	Plate PM21D	G5-G8
Cation	Palladium(II) chloride	Plate PM25D	G5-G8
Cation	Thallium(I) acetate	Plate PM22D	G9-G12
Cation	Zinc chloride	Plate PM21D	H9-H12
Chelator	1-hydroxypyridine-2-thione	Plate PM21D	D1-D4
Chelator	2,2′-Dipyridyl	Plate PM21D	A5-A8
Chelator	BAPTA	Plate PM22D	E5-E8

Chelator	EDTA	Plate PM21D	D5-D8
Chelator	EGTA	Plate PM24C	E5-E8
Chelator	Sodium pyrophosphate decahydrate	Plate PM24C	E9-E12
Other	2-deoxy-D-glucose	Plate PM23A	G9-G12
Other	3-amino-1,2,4- triazole	Plate PM22D	C1-C4
Other	4-aminopyridine	Plate PM25D	C1-C4
Other	5-fluorocytosine	Plate PM25D	G1-G4
Other	5-fluorouracil	Plate PM25D	H9-H12
Other	6-azauracil	Plate PM24C	D1-D4
Other	Amitriptyline hydrochloride	Plate PM25D	C5-C8
Other	Azaserine	Plate PM22D	F1-F4
Other	Benzamidine	Plate PM22D	G1-G4
Other	Benzethonium chloride	Plate PM23A	A1-A4
Other	Berberine	Plate PM24C	E1-E4
Other	Caffeine	Plate PM22D	A9-A12
Other	CCCP	Plate PM22D	D9-D12
Other	Cetylpyridinium chloride	Plate PM21D	C1-C4
Other	Chloroquine	Plate PM25D	H1-H4
Other	Chlorpromazine hydrochloride	Plate PM23A	A5-A8
Other	Chlortetracyclinehydrochloride	Plate PM24C	C1-C4
Other	Cinnamic acid	Plate PM25D	H5-H8
Other	Cisplatin	Plate PM24C	G1-G4
Other	Compound 48/80	Plate PM21D	E1-E4
Other	Cycloheximide	Plate PM22D	G5-G8
Other	D-serine	Plate PM22D	E9-E12
Other	Diamide	Plate PM21D	H1-H4
Other	Domiphen bromide	Plate PM21D	C5-C8
Other	Doxycycline hyclate	Plate PM23A	B9-B12
Other	FCCP	Plate PM22D	E1-E4
Other	Fluorodeoxyuridine	Plate PM25D	D9-D12
Other	Fumaric acid	Plate PM25D	F9-F12
Other	Glycine hydrochloride	Plate PM23A	C1-C4

Other	Glycine hydroxamate	Plate PM22D	B5-B8
Other	Guanidine hydrochloride	Plate PM21D	A1 - A4
Other	Hydroxylamine hydrochloride	Plate PM23A	C5-C8
Other	Hydroxyurea	Plate PM25D	A1-A4
Other	Isoniazid	Plate PM24C	F1-F4
Other	L-arginine hydroxamate	Plate PM22D	B1-B4
Other	L-aspartic acidhydroxamate	Plate PM21D	C9-C12
Other	L-glutamic acid hydroxamate	Plate PM22D	A1-A4
Other	Malic acid	Plate PM25D	F1-F4
Other	Methyl viologen dichloride hydrate	Plate PM24C	F5-F8
Other	Miconazole nitrate	Plate PM24C	H9-H12
Other	Myclobutanil	Plate PM22D	H9-H12
Other	Niaproof	Plate PM25D	A9-A12
Other	Nystatin	Plate PM21D	B1 - B4
Other	Poly-L-lysine hydrochloride	Plate PM23A	C9-C12
Other	Promethazine	Plate PM21D	A9 - A12
Other	Propiconazole	Plate PM24C	H1-H4
Other	Protamine sulfate	Plate PM21D	B9 - B12
Other	Sodium azide	Plate PM23A	F1-F4
Other	Sodium caprylate	Plate PM23A	F5-F8
Other	Sodium salicylate	Plate PM25D	E1-E4
Other	Succinic acid	Plate PM25D	E5-E8
Other	Tartaric acid	Plate PM25D	F5-F8
Other	Tetrazolium violet	Plate PM25D	B5-B8
Other	Thiourea	Plate PM21D	H5-H8
Other	Urea hydrogen peroxide	Plate PM22D	D5-D8
