# Supplementary material for: High-Throughput Chemical Screening Identifies Compounds that Inhibit Different Stages of the Phytophthora agathidicida and Phytophthora cinnamomi Life Cycles
Source: Front Microbiol. 2017 Jul 19;8:1340. doi: 10.3389/fmicb.2017.01340 (PMC5515820; doi:10.3389/fmicb.2017.01340)
Supplement: Supplementary file 2 [file Table_2.pdf]

Supplementary Material

High-throughput chemical screening identifies compounds that inhibit different stages of the *Phytophthora agathidicida* and *Phytophthora cinnamomi* life cycles

Scott A. Lawrence, Charlotte B. Armstrong, Wayne M. Patrick and Monica L. Gerth*

***Correspondence:** Corresponding Author: monica.gerth@otago.ac.nz

Supplementary Table 2. Chemical analysis of the pond water used for *P. agathidicida* sporangium production. Analysis was carried out by Citilab, Dunedin, New Zealand.

**Chemical**	**Concentration (g/m^3)**
Bicarbonate	65
Calcium (dissolved)	9.54
Magnesium (dissolved)	4.56
Nitrate	0.24
Nitrate-N	0.05
Nitrite	<0.1
Nitrite-N	<0.05
Phosphate	<0.1
Phosphate-P	<0.05
Potassium (dissolved)	1.78
Sulfate	1.0
